# Parenting experiences and outcomes among former adolescent mothers: A mixed methods study

**DOI:** 10.1371/journal.pone.0303119

**Published:** 2024-05-15

**Authors:** Serena Cherry Flaherty, M. Tish Knobf, Margaret L. Holland, Arietta Slade, LaRon Nelson, Lois S. Sadler

**Affiliations:** 1 Yale University School of Nursing, Orange, CT, United States of America; 2 Yale Child Study Center, New Haven, CT, United States of America; University of Eswatini, SWAZILAND

## Abstract

The purpose of this explanatory sequential mixed methods study was to examine parenting outcomes and experiences over time among marginalized adolescent mothers enrolled in randomized clinical trials (RCT) between 2002 and 2016 testing Minding the Baby® (MTB), an early home visiting program. The quantitative phase examined associations between measures of maternal experiences and parenting outcomes from 71 participants 2–8 years since RCT completion. MTB mothers reported less hostile parenting and fewer child behavior problems. The sequential qualitative phase involved interviews with a subsample (*n* = 31) and revealed six themes about their personal and parenting maturation. Through integration of quantitative and qualitative data, we generated metainferences, revealing a nuanced understanding of participants’ experiences. Integrated findings revealed the complex personal and parenting experiences among former adolescent mothers during their developmental phases of emerging and early adulthood. Findings inform clinical and research approaches to promote personal growth and positive parenting outcomes over time among women who began childbearing in adolescence.

## Introduction

Rates of adolescent pregnancy and childbearing are declining in the United States, yet still there are approximately 147,000 children born to adolescents ages 15–19 each year [1]. Decades of research highlight adverse outcomes for adolescent parents and children [2], including truncated education, delayed financial independence, poor mental health, and insensitive parenting behaviors [3–6]. However, with adequate support from family and specialized programs, young families can have more positive health and developmental outcomes. Early home visiting (EHV) programs often provide this support. Two notable EHV programs that specifically address parenting support for young families include the Nurse Family Partnership (NFP) and Minding the Baby® (MTB). NFP is a longstanding home visiting program that pairs nurse home visitors with young families and has demonstrated many health and parent-child relationship outcomes, especially for adolescent mothers and their children [7,8]. MTB is a similar intensive EHV program, beginning in pregnancy and lasting through the child’s second birthday, designed to enhance the maternal-child relationship, reflective parenting, that is the capacity for a parent to keep a child’s feelings, needs, and intentions in mind [9], and maternal and child health outcomes among first-time mothers ages 14–25 [10,11].

While there is an extensive body of research on adverse maternal outcomes among women who have a first birth in adolescence, less is known about how adolescent mothers grow and change when they enter the developmental phase of emerging adulthood. Emerging adulthood, defined as the period between ages 18–29 wherein individuals develop their identities and increased independence, is marked by many life transitions and developmental demands [12–15]. In addition to these developmental demands, former adolescent mothers simultaneously experience demands of the parenting role, although this compound developmental process has not yet been studied.

Additionally, there is little evidence about the sustaining effects of EHV programs for adolescent mothers (<age 22 at birth). In particular, less is known about whether the components of the MTB EHV program may or may not have lasting effects as adolescent mothers mature, their children grow and develop, and their families change over time. MTB was compared with usual women’s health and pediatric care, provided in local community-based health care centers, and tested through two randomized controlled trials (RCT) between 2002–2016. Follow-up studies have assessed ongoing effects of the MTB program [16,17]. The Early School Age (ESA) follow-up cohort study (2–8 years after RCT completion) examined the associations between maternal experiences, parenting outcomes, and intervention effects (control and intervention) [16]. These associations have not yet been explored in women who are former adolescent mothers. Additionally, the subjective perspectives of these mothers have not been explored regarding any lasting influence of EHV on their parenting.

The present study addresses the knowledge gaps related to understanding the long-term effects of an EHV intervention for adolescent mothers and their children, and mothers’ ongoing parenting experiences throughout emerging adulthood. The research questions are well-suited to a mixed methods approach because of the complexity, ongoing developmental processes, and intersectional identities of young mothers. This study provides insight into the complex experiences of parenting and adult development among former adolescent mothers. Further, it highlights implications and future directions for research, clinical practice, and policies on long-term parenting outcomes, experiences, and health equity among marginalized former adolescent mothers and their children.

### Conceptual background

**Fig 1:**
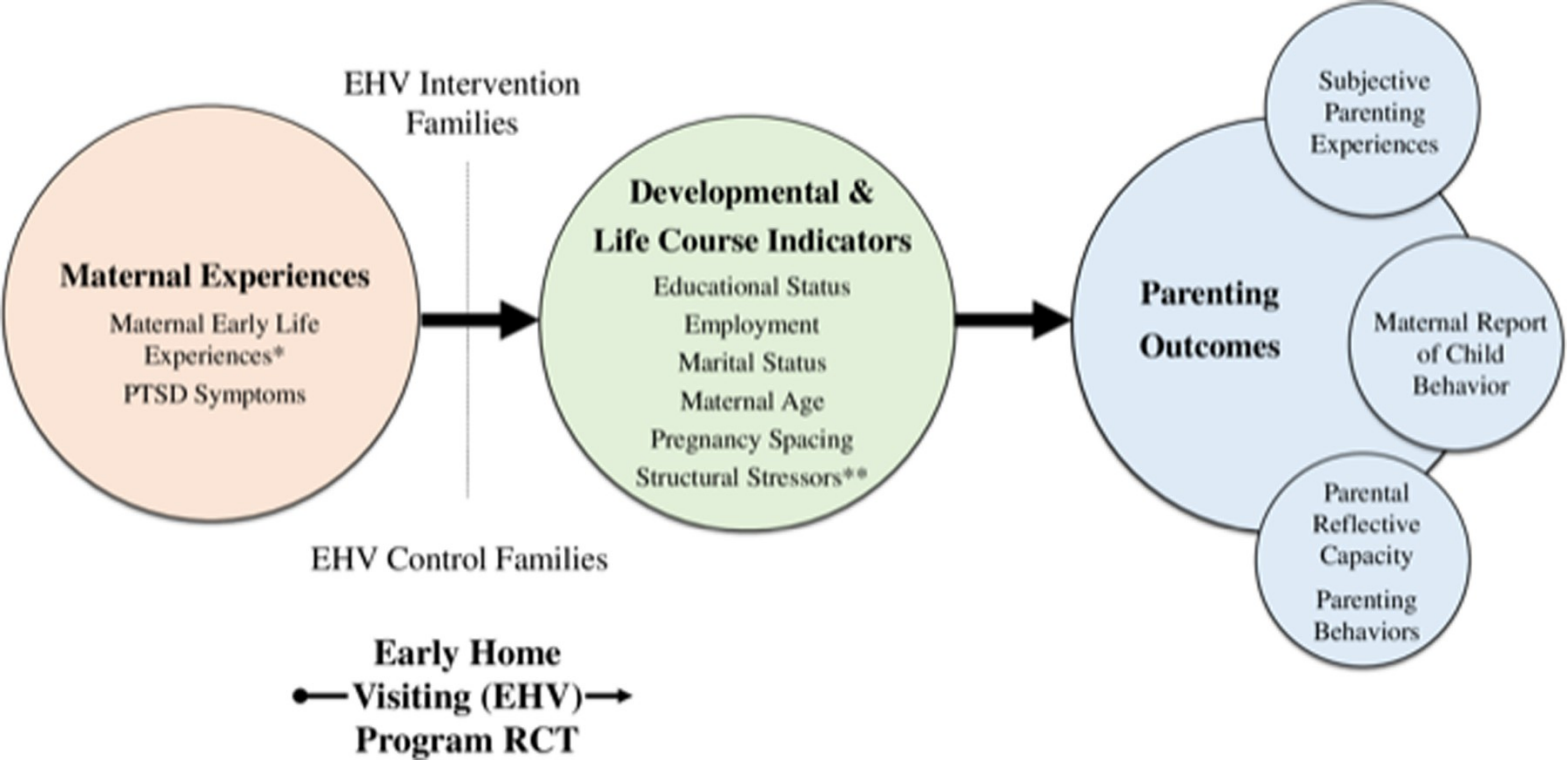
Parenting process for adolescent mothers framework. * Measured by Childhood Trauma Questionnaire Total score (5 childhood maltreatment subscales and 1 family strengths subscale). ** Structural stressors include sociodemographic factors and experience of COVID-19 pandemic.

Parenting is multidimensional and influenced by several factors, including but not limited to child development (age and behavior), parent characteristics such as personality, resources, and social support, and many additional individual contextual factors [18]. The Parenting Process for Adolescent Mothers framework (PPAM; Fig 1) was adapted from Belsky’s Determinants of Parenting Model [18]. This adapted conceptual framework illustrates the relationships among maternal experiences (contextual sources of stress and support), developmental indicators that may influence their parenting capacity, and three interrelated parenting outcomes (parental reflective capacity, parenting behaviors, maternal reports of child behavior). In addition, maternal subjective experiences of parenting may reflect many intersecting identities in the lives of young mothers. Intersectionality, a model grounded in Black feminist theory, describes the interdependence of social categories (e.g., gender, race, class) that cause inequities among groups of individuals through cultural marginalization and social oppression [19–21]. Since many adolescent mothers in the U.S. are culturally, socially, and economically marginalized, intersectionality provides an opportunity to potentially *redefine* the perceived social construct that adolescent parenthood is aberrant and *identify individual and contextual strengths* that further support the development of adolescent mothers as parents and emerging young adults [22]. Intersectionality provides a frame for integrating complex sociodemographic factors, identities, and contextual experiences (such as developmental and life course indicators in Fig 1).

### Study purpose

Guided by the PPAM framework, the purpose of this explanatory sequential mixed methods study was to examine parenting outcomes and experiences of parenting among the subsample of former adolescent mothers who participated in the MTB ESA study (control and MTB groups). This longitudinal sample, drawn from the original MTB RCTs, provided a unique opportunity to address two key research questions: (1) What are the parenting outcomes and experiences of parenting among former adolescent mothers over time as they develop through the stage of emerging adulthood, and (2) What are the group differences over time between control and MTB group participants? These research questions were addressed through three aims. Our first aim (quantitative) was to describe maternal experiences (maternal early life adversity, Post-Traumatic Stress Disorder (PTSD) symptoms) and parenting outcomes (parental reflective capacity, parenting behaviors, maternal report of child behavior) among a subsample of former adolescent mothers (<22 at RCT consent) who enrolled in the MTB ESA follow-up study while controlling for group status (MTB/control). Our second aim (qualitative) was to explore the experiences of parenting over time and the experiences of control and intervention participation in an EHV intervention RCT among former adolescent mothers. Our third aim (mixed methods) was to generate a comprehensive understanding of relationships between maternal experiences, parenting experiences and outcomes, and MTB experiences among former adolescent mothers through integration of quantitative and qualitative data.

## Materials and methods

### Design

**Fig 2:**
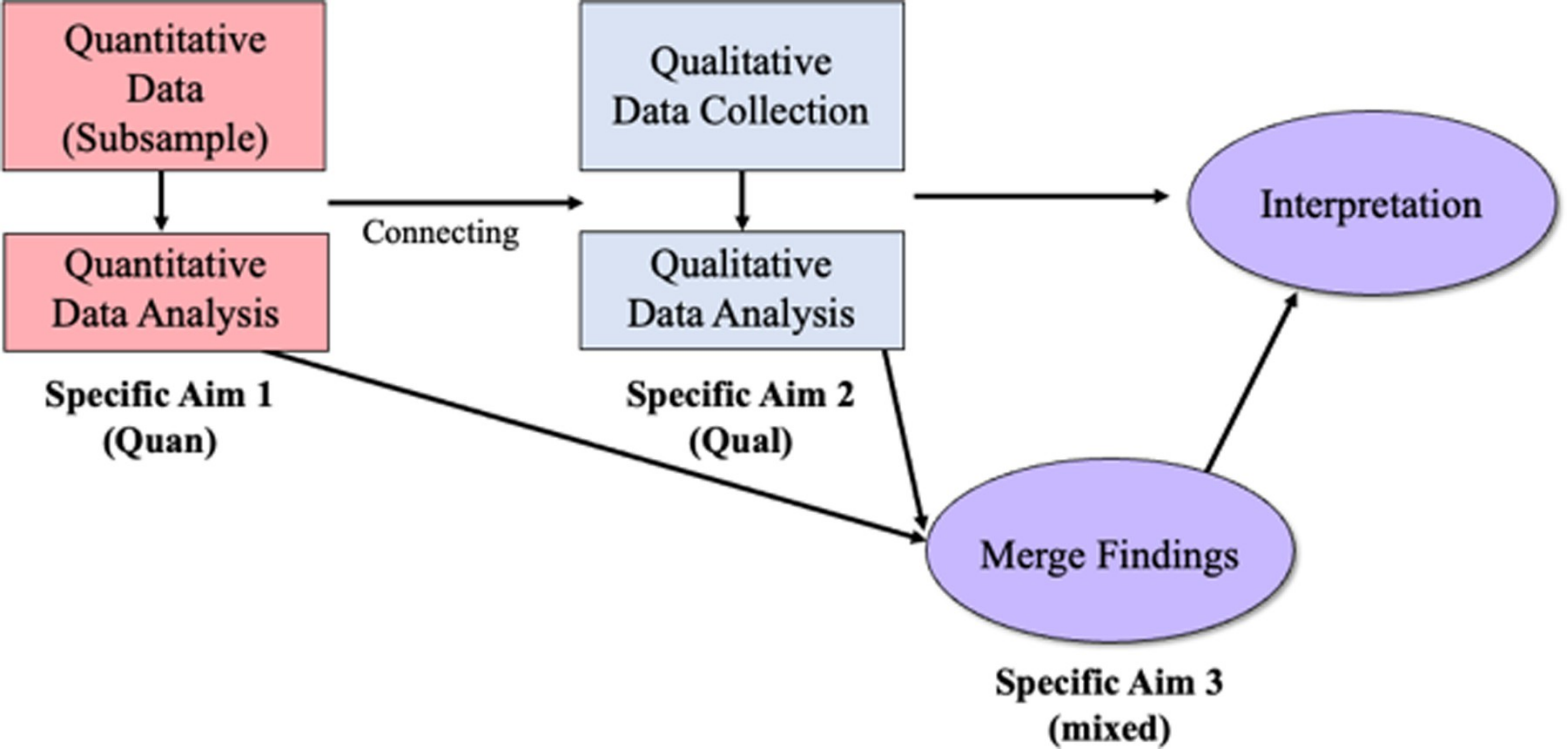
Explanatory sequential mixed methods design.

The study design was explanatory sequential mixed methods (Fig 2) [23–25]. For the quantitative phase, we conducted a cross-sectional secondary data analysis of maternal experience and parenting outcome measures from the MTB Early School-Age (ESA) cohort, within a subsample of 71 mothers.

The quantitative analysis and findings guided the qualitative phase by directing sampling and augmenting the interview guide. Stratified purposive sampling was used, a method of maximum variation sampling where participants from both control and MTB groups were purposively recruited based on quantitative findings and demographic characteristics [26]. For the qualitative phase, semi-structured interview data were collected from participants regarding their experiences as parents and young adults. Integration of the quantitative and qualitative data was conducted to gain greater insight into parenting experiences and outcomes among former adolescent mothers.

### Quantitative phase

#### Sample and setting

The quantitative phase of this study included a subsample of the MTB ESA follow-up cohort study (2016–2018). In the ESA study, mothers with index children ages 4–10 from the original MTB RCT sample who resided in or around a northeastern city, were invited to participate in a cross-sectional study in which researchers examined associations between past maternal experiences, maternal health and mental health, and caregiving patterns among RCT control (*n* = 54) and MTB (*n* = 43) group participants (ages 14–26 at RCT enrollment) [16,27]. The sample of the ESA study is described in a separate manuscript [16]. The subsample for the quantitative secondary analysis included participants in the ESA follow-up cohort study who were less than 22 years old at the time of RCT consent (*n* = 39 control; *n* = 32 MTB).

#### Procedure

After obtaining approval from the Yale University Institutional Review Board (IRB), secondary quantitative analysis was conducted with the subsample of former adolescent mothers on selected variables noted below and presented in S1 Table.

*Variables & measures*. **Social & Demographic Characteristics.** Maternal demographic data included mothers’ age at the beginning of the original RCT, age at ESA data collection, race and ethnicity, educational level (highest grade completed), employment status, number of pregnancies and births, use of public assistance programs (reflecting socioeconomic status) and original RCT group assignment [16,28].

#### Maternal Experiences

Mothers in the ESA study completed The Childhood Trauma Questionnaire-Short Form (CTQ-SF), a 28-item retrospective self-report measure that includes a total score, five subscales of childhood maltreatment (physical abuse, emotional abuse, sexual abuse, physical neglect, and emotional neglect) and one subscale of family strengths scored such that lower scores indicate more family strengths [29]. All mothers completed The PTSD Checklist-Civilian Version (PCL-C), a 17-item self-report questionnaire to assess symptom experiences of PTSD over the past month resulting from any trauma [30]. Both measures demonstrated acceptable reliability and validity across diverse community samples of adults and adolescents [16,29].

#### Parenting Outcomes

The Parental Reflective Functioning Questionnaire (PRFQ) is an 18-item questionnaire to assess reflective parenting, a parent’s capacity to imagine a child’s mental state, thoughts, and feelings [31]. Although the PRFQ includes three subscales (Interest and Curiosity in Mental States (PRFQ-IC), Certainty about Mental States (PRFQ-CM), and Prementalizing Modes (PRFQ-PM)), the PRFQ-CM was omitted from this study due to low reliability (α = 0.44; (16)). The PRFQ-IC assesses a parent’s attentiveness to their child’s mental states, and the PRFQ-PM assesses a parent’s inability to be reflective about their child’s mental states [32]. Mothers also completed the Parent Behavior Inventory (PBI), a 20-item self-report measure to evaluate parenting behaviors among parents of early school-age children [33], with two subscales (Supportive/Engaged and Hostile/Coercive parenting). Supportive/Engaged parenting is demonstrated by warmth and affection and Hostile/Coercive parenting behaviors may include threats and punishment. The Child Behavior Checklist (CBCL/1½-5 and CBCL/6-18) is a questionnaire for parents of children ages 1½-5 and 6–18, respectively, to describe child behavior and emotional problems [34]. The CBCL consists of a total score and two subscales that assess “internalizing” behaviors, such as mood or anxiety symptoms and “externalizing” behaviors, such as disruptive behaviors in children.

#### Quantitative data analyses

Univariate statistics were used to describe sample characteristics and study variables, including normality of data distribution, outliers, missing data, sample characteristics, independent variables (CTQ, PCL-C), and dependent variables (PRFQ-IC and PM, CBCL, and PBI).

For comparisons between the demographic characteristics of control and MTB groups, we used independent samples t-tests for normally distributed continuous variables and Mann-Whitney U for all other continuous variables. Maternal race and ethnicity were described as in the ESA study, including Black, Hispanic, and another race or ethnicity (n = 3). We used the statistically appropriate chi-square or Fischer’s exact test for categorical variables.

We conducted bivariate analyses to describe the strength (effect size) and direction of relationships between maternal experience variables and parenting outcome variables in the total sample and in each group (control/MTB). Data distributions were normal for CBCL Total Problems, CBCL Internalizing Behaviors, and the PBI Hostile/Coercive subscale. PRFQ-IC was cube transformed, and this improved distribution of the variable. CBCL Externalizing Behaviors was log transformed, and this improved distribution of the variable. Transformations did not improve the distribution of PRFQ-PM and PBI supportive/engaged variables. P-values for PRFQ-IC and CBCL Externalizing Behaviors were determined by t-tests using the transformed variable described above. P-values for the remaining non-transformed variables were determined using non-parametric tests. Spearman’s rank was calculated for all correlations.

Regression analyses were performed to examine the associations between maternal experiences and parenting outcomes; in all analyses, assumptions were tested, and where not met, transformations were performed. The regression coefficients for Interest and Curiosity subscale (PRFQ-IC) and PBI Hostile/Coercive subscale were standardized to facilitate interpretation. Prementalizing Modes (PRFQ-PM) scores and PBI Supportive/Engaged scores lacked variability and did not meet assumptions for linear regression, and for this reason they were dichotomized at the median, and logistic regressions were conducted. We conducted regression analyses controlling for group status and race and ethnicity because of the statistically significant difference on this characteristic between the groups.

*Power*. Although these associations have not previously been examined in this sample, we anticipated small (r = .1) to moderate (r = .3) effect sizes based on related analyses [16,27]. Using correlations from the ESA study between CTQ scores and PBI (Supportive/Engaged behaviors; r = -.29) and CTQ and PRFQ (Prementalizing Modes; r = .39), the sample size (n = 71), and α = .05, the estimated power for detecting significant associations in Aim 1 was between .70 and .93. These power calculations were computed based on effect sizes from the full ESA sample which included older mothers, but as 73% of the full sample were included in this study, effect sizes in the subsample were not likely to be substantially different.

### Qualitative phase

#### Procedure

The recruitment period for the qualitative phase was July 6, 2021, to September 8, 2021. Eligible participants were contacted through email and text messages that contained a study flyer. Recruitment for the qualitative phase is described fully in a methods manuscript related to this study [35]. The Yale University IRB granted a waiver of written consent as all interviews were conducted virtually and the study met criteria for this waiver. Since COVID-19 physical distancing recommendations remained in effect for the study period, all participants were interviewed using Zoom, a secure synchronous videoconferencing software program [36], which is compatible with smartphones and increasingly used as an acceptable alternative method of data collection [37,38]. Participants elected audio or video functionality on Zoom, and in all cases only audio was recorded. All interviews were conducted by the interviewer alone using an institutional Zoom account in a private office; participant location was not disclosed to the interviewer. In some cases, mothers had their young children in their care at the time of the interview. The interviewer reviewed the consent form with each participant over Zoom, and participants had the opportunity to ask any questions. If they agreed to participate in the study, the verbal consent was witnessed by the interviewer over Zoom and documented by the interviewer on the IRB-approved consent form, as approved by the IRB. In keeping with the procedures of prior follow-up MTB studies, participants received a $50 e-gift card for participation in the qualitative phase of the study.

The qualitative phase included a demographic survey and semi-structured interviews. The Principal Investigator (PI), a female doctoral candidate with extensive coursework and training in qualitative research, conducted all interviews in English. An initial interview guide was developed, pilot tested with members of the MTB team, and refined based on the quantitative findings. The PI had no known established relationships with participants, although she had worked as a research assistant on the study 2005–2006, and in one case she was recognized as a study team member by a participant. At the outset of the interview, the PI described her professional background as a pediatric nurse practitioner, and reasons for conducting the study. In cases where participants had their cameras on, the interviewer recorded field notes, which included background, notable distractions, and participant non-verbal cues including postures, gestures, and tone. These methodological reflections are described in a separate manuscript [35].

The updated demographic information was collected via a secure, institutionally licensed, self-administered online Qualtrics questionnaire (accessible via smartphones) at the time of the qualitative interview [39], and included educational status, marital status, employment status, sources of health insurance (public or private, as a general proxy for socioeconomic status), number of pregnancies and births, and fathers’ involvement.

The qualitative study design was interpretive description, an inductive approach for exploring experiences within the context of complex clinical problems [40,41]. An initial interview guide was developed, pilot tested, and refined based on the quantitative findings (S2 Table). Data collection continued until saturation of themes was determined [42], when the PI and a second researcher (L.S.) reached consensus [43]. Additional data sources included field notes, and personal reflections of the researcher and team members. The control and MTB group participants were asked the same interview questions except MTB participants were asked questions about their experiences of the MTB program.

#### Qualitative data management and analysis

Interview audio recordings were de-identified, transcribed verbatim by a professional transcriptionist, and imported into *Atlas*.*ti Version 9*.*1*.*3*© (Berlin, Germany). Transcripts were coded and thematically analyzed through a process that included data organization, immersion in the data, coding, identifying themes, writing analytic memos, considering alternative themes or understandings, and presenting the findings. The two coders (S.F. and L.S.) read all transcripts and inductively coded to develop initial codes, followed by the development and testing of the coding structure. When coding agreement on transcripts reached 80% or greater based on coding agreements relative to total coding decisions, the PI coded remaining transcripts. Notes on code groups supported the development of themes and subthemes. Codes were linked in networks to affirm identification of central themes, guided by the study’s conceptual framework, emerging adult development, and intersectionality. Descriptive participant profiles were compiled for all 31 participants, incorporating demographic information and brief summaries for each theme and subtheme. This process allowed for re-immersion in the data and aided in verifying the thematic development. Member checking with participants was done by distributing a summary of findings to participants to elicit feedback, and memos throughout the coding and mapping processes were recorded and incorporated into the analysis. The PI maintained a research log to provide an audit trail. Rigor in the qualitative phase was established by using thick description and inter-coder agreement to ensure dependability, and credibility was enhanced by member checking, multiple data sources (former adolescent mothers who did and did not receive an EHV intervention), and reporting disconfirming evidence [23]. Rigor was also ensured by reporting in accordance with the COREQ (Consolidated criteria for Reporting Qualitative Research Checklist) (S3 Table) [44].

### Mixed methods phase

#### Procedure

Consistent with mixed methods design, integration occurred through two distinct approaches: (1) *connecting* and (2) *explaining* [23,26]. First, data integration through *connecting* occurred between the phases by building the qualitative sample and interview protocol informed by results from the quantitative analysis. Second, data integration through *explaining* occurred following quantitative and qualitative data collection to determine if there was convergence or divergence between quantitative measures of parenting outcomes (PRFQ, PBI, and CBCL) and qualitative descriptions of participants’ experiences of parenting. Finally, merged quantitative and qualitative data were integrated in two figures to jointly display the integrated results [23].

#### Mixed methods data management and analysis

This phase of integration followed the steps outlined by Fetters [24], including linking constructs from the quantitative data to the qualitative data. Quantitative data (focusing on statistically significant findings) and qualitative data (extracting summary statements for each theme from the participant profiles) were combined in two Excel documents (one for each research question). The PI drew metainferences from the quantitative and qualitative data to inform an interpretation of the mixed data. Notes on the integration phase informed the development of two joint displays which were reviewed and refined by L.S. and a third expert in mixed methods research (M.T.K.).

## Results

### Quantitative phase

Characteristics of the sample are described in Table 1. The average age at mothers’ consent was 25.4 ± 2.8 years, with no statistically significant differences in age between groups (control/MTB). The mean age of children was 6.8 ± 1.8 years. In the sample, 23 participants (32%) reported their race as Black, while 45 participants (63%) reported their ethnicity as Hispanic (Hispanic white (*n* = 18), and Hispanic other (*n* = 27)). Mothers with less than high school education comprised 43% of the sample and 91% reported receiving public assistance.

**Table 1:** Demographic characteristics of the sample.

	Quantitative Sample				Qualitative Sample
	Total (*n* = 71)	Control Group (*n* = 39)	Intervention Group (*n* = 32)		Total (*n* = 31)
Characteristic	Mean (*SD*)	Mean (*SD*)	Mean (*SD*)	*p* value	Mean (*SD*)
Mother’s age at RCT consent (years)	18.3 (1.9)	18.2 (1.9)	18.4 (1.8)	.70	18.3 (1.6)
Mother’s age (years) time of study^a^	25.4 (2.8)	25.2 (2.9)	25.7 (2.6)	.43	29.7 (2.5)
Index child’s age (years) time of study	6.8 (1.8)	6.8 (2.0)	6.9 (1.6)	.48	10.7 (1.9)
Time to follow up (years since RCT)	4.8 (1.8)	4.8 (2.0)	4.9 (1.6)	.48	8.7 (1.9)
	*n* (%)	*n* (%)	*n* (%)	*p* value	*n* (%)
Maternal Race/Ethnicity^b^					
Black	23 (32)	17 (43)	6 (18)	.02*	12 (38)
Hispanic	45 (63)	20 (51)	25 (78)		17 (54)
Another	3 (4)	2 (5)	1 (3)		2 (6)
Education				.52	
Less than high school	30 (42)	17 (43)	13 (41)		6 (19)
Completed high school	20 (28)	13 (33)	7 (22)		4 (12)
Some post high school training/Some college	20 (28)	9 (23)	11 (35)		13 (41)
Completed college or some graduate school	-	-	-		8 (25)
Marital Status				.54	
Single or Divorced/Separated	44 (62)	26 (66)	18 (56)		17 (54)
Married or Living Together	27 (38)	13 (33)	14 (43)		14 (45)
Employment Status at Time of ESA Study				.32	
Not working outside home	22 (31)	11 (28)	11 (34)		8 (25)
Working part-time	22 (31)	15 (38)	7 (31)		7 (22)
Working full-time	27 (38)	13 (33)	14 (43)		16 (51)
Socioeconomic Status^c^				.69	
Receiving public assistance^d^	64 (91)	35 (89)	29 (93)		-
Receiving no public assistance	6 (8)	4 (10)	2 (6)		-
State healthcare plan (Medicaid)	-	-	-		23 (74)
Private/employer plan	-	-	-		7 (22)

*Note*. Information about education and socioeconomic status were collected with different survey questions in the quantitative and qualitative sample.

^a^ Independent samples t-test; Mann-Whitney U for all other continuous variables.

^b^ p-value for chi-square test was determined using Black and Hispanic categories; Another was excluded from chi-square test due to small cell size.

^c^ Fisher’s exact test; chi-square test for all other categorical variables.

^d^ Public assistance includes enrollment in one or more of the following public assistance. programs: Supplemental Nutrition Assistance Program (SNAP), Temporary Assistance for. Needy Families (TANF), Special Supplemental Nutrition Program for Women, Infants, and Children (WIC), or Medicaid of Children’s Health Insurance Program.

*p < .05.

Unadjusted study variable characteristics for the total sample and for MTB and control groups are presented in S4 Table. There were statistically significant differences between groups for Hostile/Coercive parenting (*p* = .009) and CBCL Externalizing Behaviors (*p* = .03) and CBCL Internalizing Behaviors (*p* = .03) such that the MTB group demonstrated lower Hostile/Coercive parenting and Externalizing and Internalizing Behavior scores.

Correlations informed the regression analyses (S5 Table). Regression results from the PRFQ-IC and PRFQ-PM subscales are presented in Table 2. Maternal early life adversity was statistically significantly associated with lower Interest and Curiosity (ß = -0.29, *p* = .04), when controlling for PTSD symptoms, group status, and race and ethnicity. PTSD symptoms, however, were associated with an increase in Interest and Curiosity (ß = 0.34, *p* = .01), when controlling for maternal early life adversity, group status, and race and ethnicity, and this was not in the expected direction. There were no statistically significant associations between maternal early life adversity and PTSD symptoms and Prementalizing.

**Table 2:** Adjusted regression analyses for maternal characteristics and reflective parenting.

Variable	PRFQ–Interest and Curiosity^a^		PRFQ-Prementalizing Modes High vs. Low (ref)^b^	
	*n* = 67		*n* = 68	
	β (*SE*)	*P*	OR (95% CI)	*p*
CTQ Total Score	-0.29 (0.13)	.04*	1.02 (0.98, 1.07)	.31
PCL-C	0.34 (0.13)	.01*	1.02 (0.97, 1.06)	.54
Intervention (vs. Control)	-0.10 (0.11)	.36	0.40 (0.13, 1.29)	.12
Black (vs. Hispanic)	0.09 (0.11)	.41	0.38 (0.11, 1.23)	.11

Note.

^a^ Linear regression with standardized outcome variable.

^b^ Logistic regression with outcome variable categorized at median split.

*p < .05

** *p* < .01

*** *p* < .001.

Regression analyses results for maternal characteristics and PBI subscales (Supportive/Engaged and Hostile/Coercive) are presented in Table 3. MTB mothers were not statistically significantly more likely to have Supportive/Engaged parenting behaviors; however, being in the MTB group was statistically significantly associated with lower levels of Hostile/Coercive parenting behaviors (ß = -0.36, *p* = .005).

**Table 3:** Adjusted regression analyses for maternal characteristics and parenting behaviors.

Variable	PBI–Hostile/Coercive^a^		PBI–Supportive/Engaged High vs. Low (ref)^b^	
	*n* = 68		*n* = 68	
	β (*SE*)	*p*	OR (95% CI)	*p*
CTQ Total Score	-0.15 (.15)	.32	0.98 (0.94, 1.02)	.23
PCL-C	0.27 (0.15)	.07	0.99 (.95, 1.03)	.54
Intervention (vs. Control)	-0.36 (0.12)	.005**	1.15 (0.40, 3.33)	.12
Black (vs. Hispanic)	-0.05 (0.12)	.67	0.73 (0.24, 2.22)	.11

Note.

^a^ Linear regression with standardized outcome variable.

^b^ Logistic regression with outcome variable categorized at median split.

*p < .05

** *p* < .01

*** *p* < .001.

Regression results for associations between maternal early life adversity and child behavior problems (CBCL Total Problems, CBCL Internalizing Behaviors, CBCL Externalizing Behaviors) are presented in Table 4. Maternal PTSD symptoms were statistically significantly associated with higher CBCL Total Problems (ß = 0.34, *p* = < .001), higher Internalizing Behaviors (ß = 0.26, *p* = .006), and higher Externalizing Behaviors scores (ß = 0.29, *p* = < .004). Mothers in the MTB group reported significantly lower CBCL Total Problems (ß = -4.61, *p* = .049), and significantly lower Externalizing Behaviors (ß = -5.89, *p* = .02).

**Table 4:** Adjusted regression analyses for maternal characteristics and child behaviors.

Variable	CBCL Total Problems		CBCL– Internalizing Behaviors		CBCL– Externalizing Behaviors	
	*n* = 68		*n* = 68		*n* = 68	
	β (*SE*)	*p*	β (*SE*)	*p*	β (*SE*)	*p*
CTQ Total Score	0.13 (0.08)	.09	0.13 (0.08)	.10	0.08 (0.08)	.35
PCL-C	0.34 (0.09)	< .001***	0.26 (0.09)	.006**	0.29 (0.10)	.004**
Intervention (vs. Control)	-4.61 (2.29)	.049*	-2.80 (2.38)	.24	-5.89 (2.48)	.02*
Black (vs. Hispanic)	-2.74 (2.39)	0.26	-1.11 (2.48)	.66	-4.40 (2.49)	.10

Note.

*p < .05

** *p* < .01

*** *p* < .001.

### Qualitative phase

The sampling strategy for recruiting participants for interviews was determined by the significant quantitative findings. Three statistically significant outcomes in the regression analyses (PRFQ-IC, PBI Hostile/Coercive, and CBCL) guided the sampling. We selected PRFQ-IC as the variable for sampling since reflective parenting was one of the core principles of the MTB intervention. We purposively sampled initially based on the median split of PRFQ-IC scores and across control/MTB groups from the quantitative sample (*N* = 71). Thirty-one participants enrolled in and completed the study; one participant who responded to recruitment messages declined to participate citing no interest in an interview. Among the 31 participants who completed the qualitative interviews, the sample comprised 10 MTB participants in the high PRFQ-IC score category, six MTB participants in the low PRFQ-IC score category, seven control participants in the high PRFQ-IC score category, and eight control participants in the low PRFQ-IC score category. We aimed to further sample based on self-reported race and ethnicity, as illustrated in Table 1, because of the group differences on this characteristic; however, we reached data saturation before this step. The mean maternal age at the time of the interview was 29.8 ± 2.7 years, with no difference between control and MTB group participants. The mean child age of all children in the study was 7.4 ± 4.1 years, and the mean number of children was two. Mothers who enrolled in the qualitative phase, compared with those who did not enroll, did not differ on ESA study characteristics (time from RCT to ESA follow-up, maternal age at consent, age of index child, maternal race and ethnicity, education, employment status, and socioeconomic status), except more enrolled participants (48%) reported being married or living with a partner than unenrolled participants (30%; *p* = 0.49). See Table 1 for a full description of the qualitative sample.

**Fig 3:**
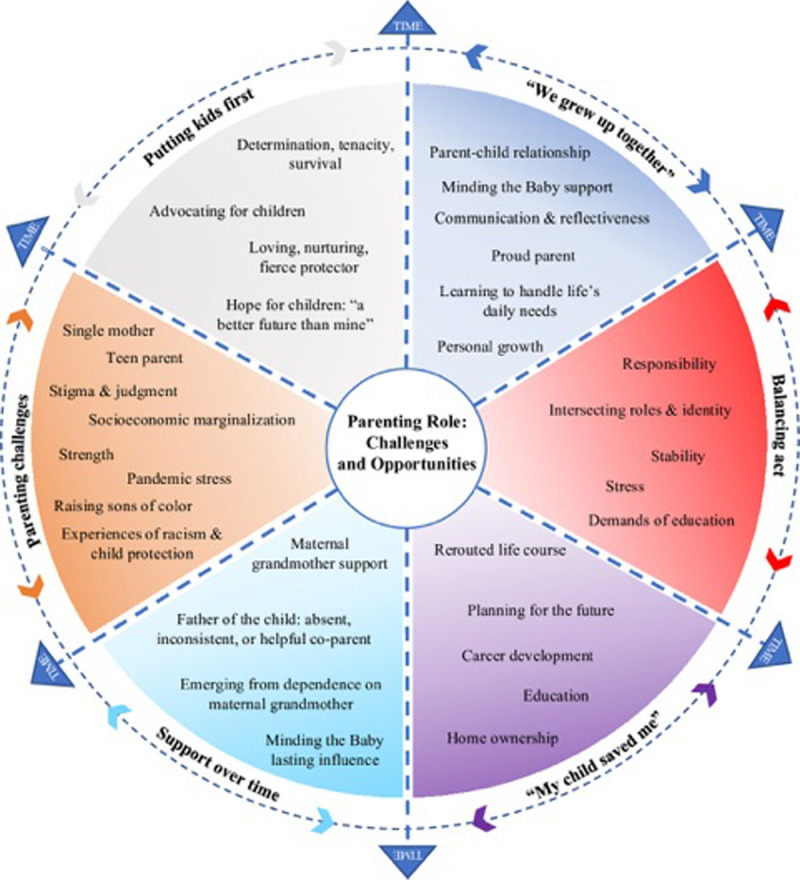
Qualitative themes.

The mean length of the interviews was 53 minutes (range 29–91). We identified six themes from the participants’ descriptions of their experiences of parenting. The overarching theme was Parenting Role: Challenges and Opportunities, which referred to the participants’ reflections on their personal and parenting growth since becoming a mother during their adolescence. Themes and subthemes are depicted in Fig 3.

#### “We Grew Up Together”

*“*We Grew Up Together*”* described the experiences of mothers on their journey of personal growth while meeting ongoing demands of parenting. Mothers spoke about how abruptly they needed to learn how to parent and manage the demands of their own lives and the needs of their children. One participant in the control group recalled, *“I needed a crash course in adulting*, *but as a mother*.*”* This theme also captured the evolution of the maternal-child relationship over time, including the tight bond mothers reported with their children. Many participants exhibited reflectiveness and described thinking about their children’s emotions, some specifically recalling reflective skills learned from MTB. One MTB participant noted, *“I learned very quickly …you can’t make any logical or rational decisions while you’re in an emotional state*…*I’m like*, *okay*, *what is this person thinkin’ right now*? *What could they possibly be feeling*?*”* Lastly, many mothers spoke about how much they matured as individuals while parenting. Some mothers described experiences of learning alongside their children, while others reflected on greater personal growth since becoming a parent through maturation, better decision making, and being role models for their children.

#### Balancing act

This theme reflected participants’ experiences juggling numerous roles and demands, including school, work, relationships, family, and parenting. Many mothers recalled shifting priorities from their own needs to those of their children, and in many cases, they recalled phasing out characteristically teenage behaviors in favor of caring for their children. They spoke about multiple intersecting identities and role responsibilities. One MTB participant said, *“I sacrifice just some me time and getting myself together for my kids*. *Going to school*, *I sacrificed a lot of sleep and sanity*, *because I was working full-time*.*”* Participants in the study experienced wide ranging stress related to balancing parenting and personal demands. Some women cited stressors in early years, and others described more recent stressors. The transition to parenting was a period of stress described by many women. Enduring stressors were described as trying to do too much, ongoing parenting demands, feeling overwhelmed with no break or support, and having added extended family caregiving responsibilities. One mother in the control group reflected:

*I had three kids*, *and I didn’t have a job*. *I was still stayin’ home with my mom*. *It was just stressful*. *I didn’t have no income*…*We were all sharin’ one bedroom*, *so that was very stressful*. *Then just dealin’ with tryin’ to understand how everybody feels*, *the kids*, *me*, *my mom at that time*, *how everybody was feelin’*. *It was stressful*.

#### “My Child Saved Me”

The theme "My Child Saved Me" captured how, in many cases, early parenting positively rerouted the mothers’ life course. They reflected how they were forced to grow up quickly, sacrifice some of their own desires and plans, and learn how to be a parent. For some it was life-saving, as described by a MTB participant:

*I was never good enough*…*everyone didn’t care about me*. *I didn’t care about anyone else*. *I was partying a lot*. *I was just hanging out*. *I was into some kind of fight or something along the line*. *There used to be about*, *maybe*, *seven girls that I used to always hang out with*. *At least four of them have been incarcerated*. *She just came at the right time*.

Parenting forced many mothers to think about planning for the future and set goals for themselves and for their children, and in many cases hopes for the future included a course that was different than their own. Many mothers spoke about how having a child affected their own educational experiences. For some, having a child propelled them to finish high school, advance to college, and in some cases, to graduate school. For others, having a baby in their teen years truncated their education.

#### Support over time

Support Over Time was identified as an important part of their growth as parents and young adults. For many, social support from their own mothers was critical, particularly throughout pregnancy and in the early years of parenting. Many participants lived with their own mothers for periods of time, depended on them for childcare, financial, and emotional support, and learned about parenting from them. Speaking about her mother’s support, one MTB participant said, *“My mom’s been my backbone throughout my whole life*.*”* However, in other cases, their own mothers’ presence was not helpful, and participants spoke about a desire to do things differently from their own mothers. As mothers grew and matured, many described emerging from dependence on their own mothers and moving toward independent, self-assured parenting.

The children’s fathers were described in one of three categories: absent, inconsistent, or helpful co-parent. Mothers described going to great lengths to emotionally protect their children when fathers were absent and inconsistent. Conversely, many mothers described very supportive and helpful partners who actively co-parented.

Additionally, some mothers spoke about the lasting influence of MTB as a source of support. Many mothers in the MTB group felt ongoing support from the connectedness in the early years, and some participants in the control group spoke about receiving letters from MTB over the years. One MTB mother spoke about feeling ongoing support from MTB, *“I feel like*, *in a certain way*, *Minding the Baby is still there*.*”*

#### Parenting challenges

Mothers described immense parenting challenges that were both pragmatic and emotional. As adolescent parents, they experienced a range of emotions, including excitement, feeling terrified and unprepared, and feeling overwhelmed by the responsibilities of parenting. One MTB mother said, *“It was hard…I knew I had to change my lifestyle*, *but I was not expecting all this*, *all this hard part about being a mom*, *a teen mom*, *and having no work*, *having no income*.*”* Many participants identified as single mothers and noted the stress and hardships they experienced as a single parent, including financial stress, lack of physical and emotional support, and day-to-day parenting stress. One MTB mother described her experience as a single parent: *“I try to keep my emotions together*. *It can get hard sometimes ’cause like*, *especially when you feel like a single mom*, *you feel like the whole world’s on your back*.*”* Stigma and judgment about being an adolescent parent were described by many mothers who experienced socioeconomic marginalization and lacked jobs with sustainable sources of income. Many mothers also spoke about stress from their experiences of racism, noting a vigilance about protecting their children from racism. Many mothers raising Black sons described exceptional fear for their children’s safety and future. Others did not report experiences of racism but were thoughtful about teaching their children about racial justice. Despite all the challenges, many mothers demonstrated enduring strength.

#### Putting kids first

Participants described their sacrifices in Putting Kids First. They were forced, in many cases, to change their plans to be able to prioritize supporting their family above all else. In their descriptions of parenting, the tone was one of fierce determination, tenacity, and survival. Demonstrating unwavering determination to protect her child, one MTB participant said, *“I didn’t care if I had go through hell and back*. *She was gonna get what she needed*. *It was just I don’t know*, *just instinct*. *It’s just like a mama bear protecting her cub*.*”* Many participants spoke about their unconditional love for their children, making time to be present with their children to nurture them, and described themselves as fierce protectors of their children–protecting them emotionally, as well as from through physical dangers and the harsh world around them. When thinking about the future, mothers held hope for their children–that they would have lives of health and happiness, reach educational milestones, and develop careers. This was often described as “a better future than mine.”

### Integration

**Fig 4:**
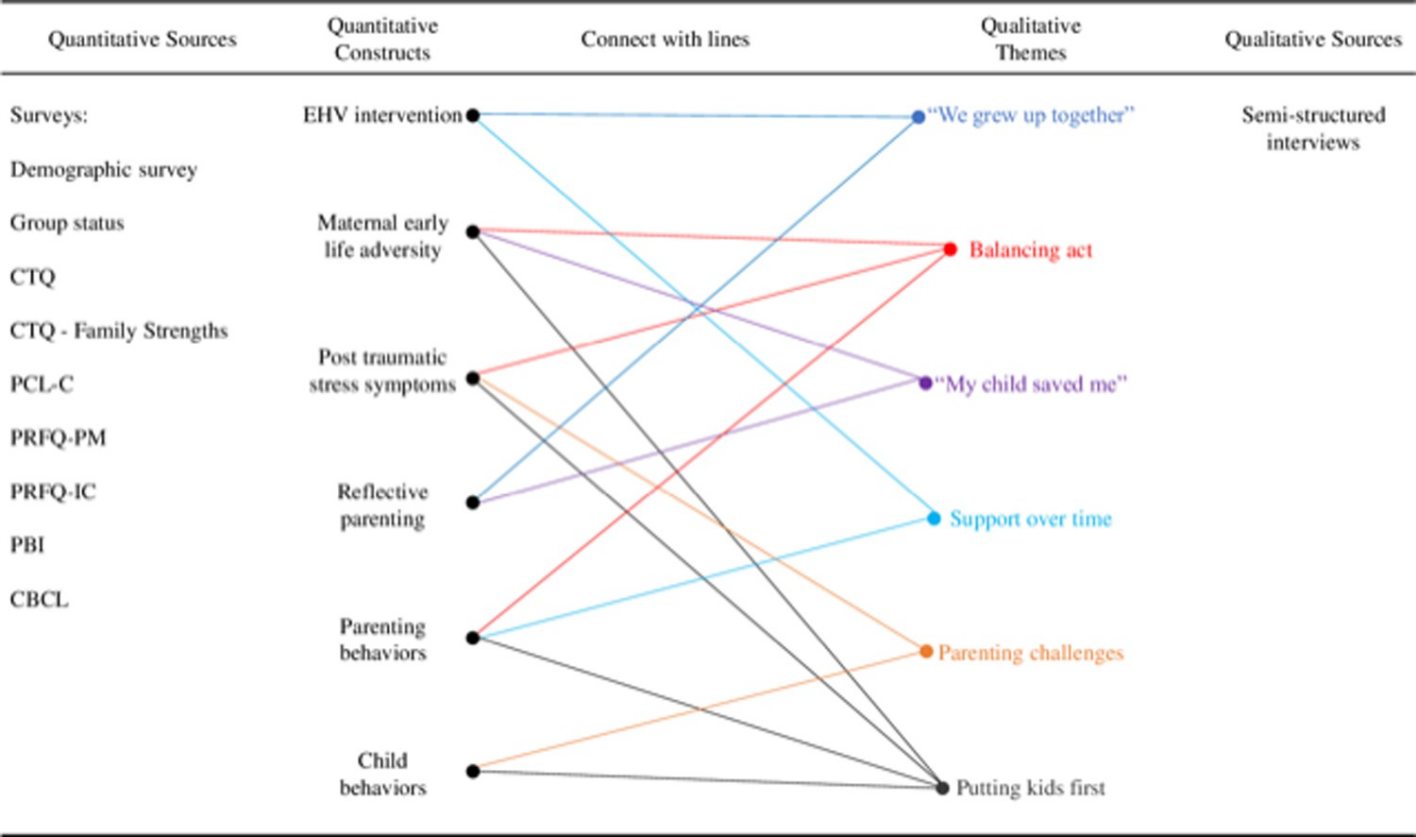
Linking quantitative constructs and qualitative themes.

**Fig 5:**
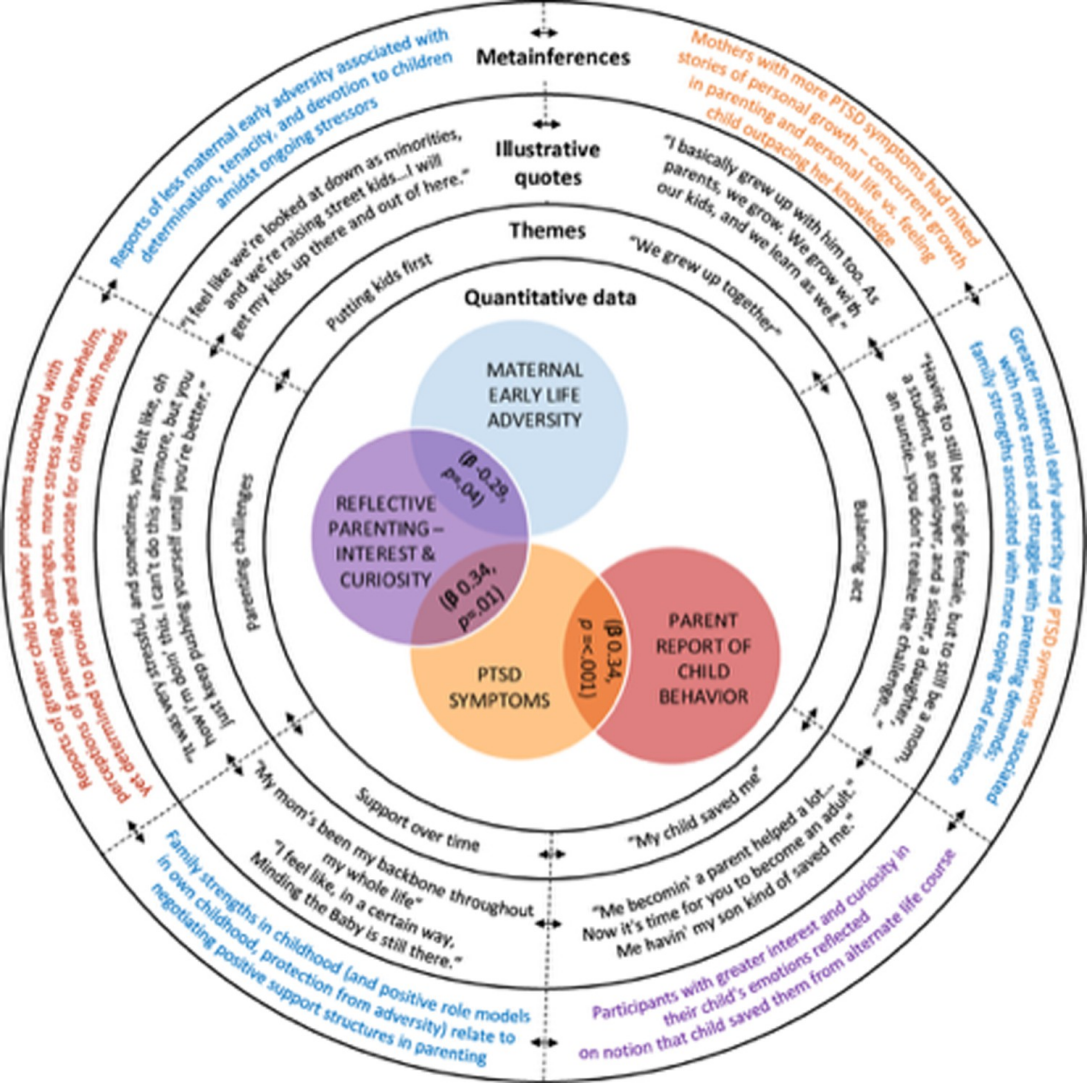
Joint display–former adolescent mothers’ experiences through emerging adulthood.

**Fig 6:**
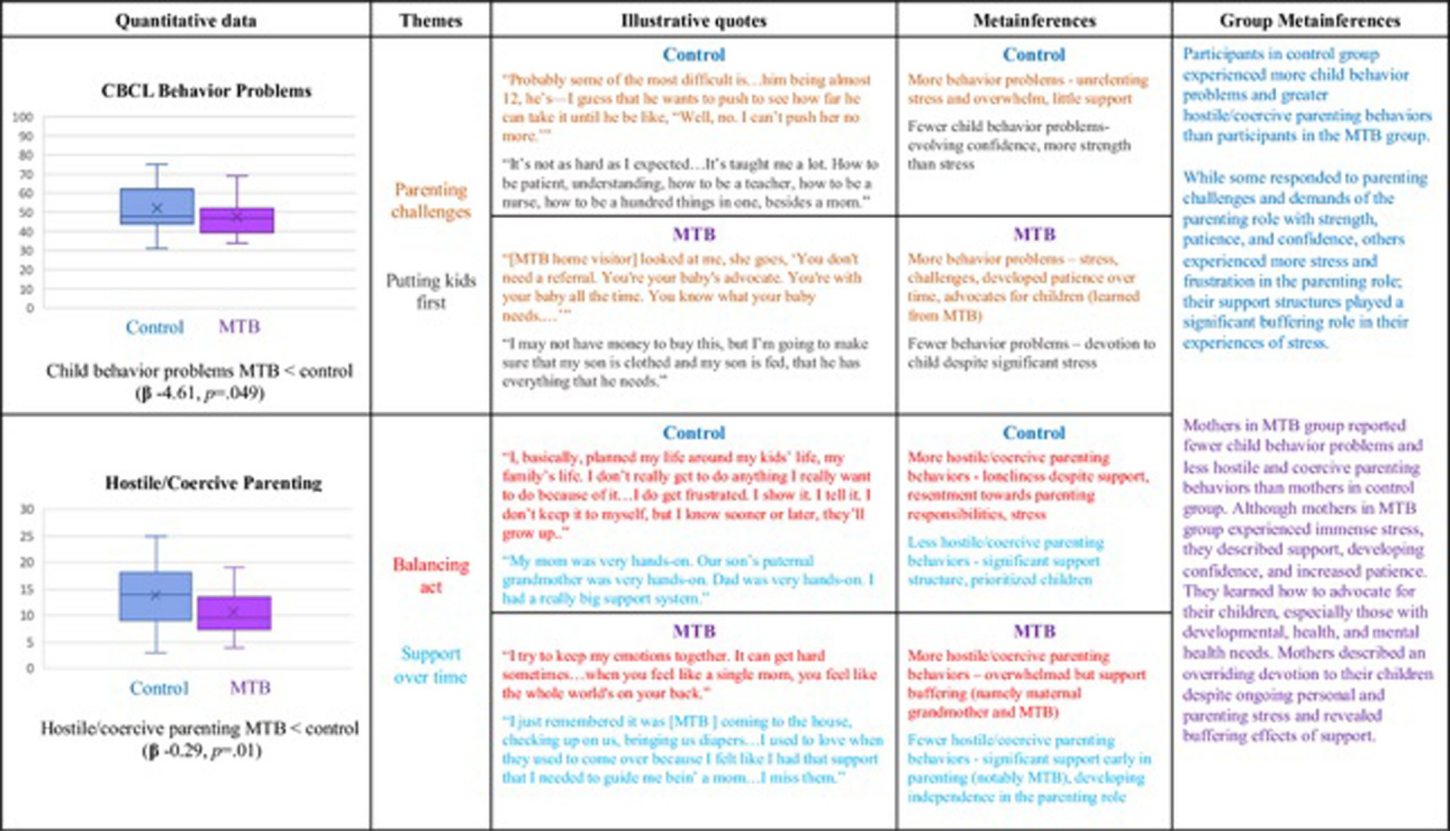
Joint display–MTB and control group differences.

The process for linking quantitative and qualitative constructs is displayed in Fig 4. Joint displays addressing the research questions are displayed in two separate figures: (1) participants’ parenting experiences through emerging adulthood (Fig 5) and (2) the control and MTB group differences (Fig 6).

The circular joint display in Fig 5 was adapted from Bustamante [45] and represents the experiences of parenting through emerging adulthood. The Venn diagram at the center displays the significant relationships from our quantitative findings regarding maternal experiences (maternal early life adversity and PTSD symptoms) and parenting (reflective parenting and parent report of child behavior) in the sample. The concentric circles that frame the central quantitative results include the qualitative themes, illustrative quotes, and metainferences that emerged from the mixed data. Bidirectional arrows around the circle indicate the interplay among each of the themes. The metainferences link the significant quantitative findings with qualitative themes (demonstrated by illustrative quotes) and are presented in the outer ring. Mothers who experienced more stress, including reported early life adversity or PTSD symptoms, had more difficulty managing ongoing parenting stresses. Mothers who described more positive parenting experiences not only experienced fewer early adversities but had support that reduced potential overwhelming experiences of parenting. The mixed methods analyses did not reveal a direct link between quantitative measures of maternal early life adversity and PTSD symptoms and descriptions of personal growth and development. In some cases, irrespective of maternal history, mothers described how much they had grown personally in ways that are consistent with emerging adult identity development. In other cases, women described experiences of growing up alongside their children, in ways that reflected a pause in their own personal growth and development. This joint display depicts the intersectional experiences, competing identities, and multiple role responsibilities experienced by former adolescent mothers as they continued to mature as parents and individuals.

Fig 6 includes quantitative data, qualitative data, illustrative quotes, and metainferences that depict the group differences with respect to parenting experiences among participants who were part of the original RCT control and MTB groups. Significant findings demonstrating a positive intervention effect for maternally reported child behavior and parenting behaviors are represented in box and whisker plots. Each quantitative variable was linked with qualitative themes, based on linked constructs. Illustrative quotes aided in drawing metainferences for each parenting outcome, and subsequently for each group. We found that while mothers in the control group reported more child behavior problems and greater hostile and coercive parenting behaviors than participants in the MTB group, they described varying responses to the challenges; some portrayed strength while others described being more overwhelmed and frustrated. Mothers in the MTB group reported fewer child behavior problems and less hostile and coercive parenting behaviors yet still described experiences of stress associated with parenting. Despite the many challenges, mothers in both groups revealed unwavering determination to love, nurture, and provide for their children.

## Discussion

The results of this mixed methods study provide a nuanced and developmental understanding of parenting experiences among former adolescent mothers. The participants in the study comprised primarily racially minoritized mothers of Hispanic ethnicity and Black race, many of whom experienced socioeconomic marginalization and high levels of self-reported early adversity and ongoing stress, contributing to the complexity of their experiences. We synthesized new knowledge in two key under-researched areas: (1) adolescent mothers’ parenting and emerging adult developmental experiences over time, and (2) ongoing parenting experiences of participants who received the MTB EHV intervention compared with those in the control group in the original RCT.

### Experiences of parenting from adolescence into emerging adulthood

Many mothers who have a first birth in adolescence face significant personal, contextual, and parenting stressors and marginalization [46,47], and adverse outcomes associated with early parenting have been well-documented (2). However, these mixed methods findings demonstrated that parenting outcomes and experiences for adolescent mothers were not altogether negative. In this study, we add to the literature by describing the complex and unique factors including the relationship between maternal early life adversity and mothers’ responses to parenting challenges, mothers’ reflectiveness about their personal growth and identity formation, and the role of social support. These main findings may serve to help guide future tailored interventions to support adolescent mothers not only through the perinatal and early childhood period, but in their personal growth and parenting role over time.

In contrast with the predominant literature describing links between maternal early life adversity and negative outcomes in adolescent pregnancy and parenting [48–50], findings from this study suggested that early adversity and mothers’ ongoing parenting experiences as well as their capacity to manage parenting challenges are related in much more subtle ways than have previously been reported in the literature. Metainferences revealed the complexities underlying the personal and parenting experiences of former adolescent mothers, and the strong influence of stress. When mothers experienced greater stress (both in the form of early life adversity and ongoing stress-related symptoms), parenting challenges were more overwhelming. Conversely, mothers who reported less early adversity described steadfast determination and tenacity in providing and caring for their children with less struggle, despite many ongoing stressors. Additionally, mothers who had described family strengths in childhood (e.g., family closeness, caregiving, and love) and social support, described more positive parenting perspectives, less stress, and were less overwhelmed. This suggests that adolescent mothers who experience adversity and have fewer family strengths may benefit from ongoing interventions to promote protective factors and provide support in parenting and young adult growth.

There is evidence that reflective parenting is diminished among mothers who reported experiences of childhood adversity and trauma, though not specifically with samples of adolescent mothers [51–54]. Although there were statistically significant associations in this study between early adversity, PTSD symptoms and PRFQ Interest and Curiosity, the effect sizes were small. We also discovered a positive association between PTSD symptoms and PRFQ Interest and Curiosity. The qualitative findings revealed that mothers who described more PTSD symptoms also described being very protective parents, often conveying hypervigilant thoughts and behaviors related to their children’s safety and exposure to the world. These findings were consistent with previous studies in adult populations, suggesting that exposure to trauma with persisting symptoms is associated with hypervigilance (a symptom of PTSD) in the parenting role [55]; however, larger studies are needed to further examine these relationships specifically among former adolescent mothers.

In developed economies, emerging adulthood generally represents the period when individuals become physically and financially independent from their parents or guardians, attend college, vocational training, or enroll in the military, marry, have children, and begin careers [12,13]. However, depending on individual and cultural contexts, not all individuals may follow this expected trajectory [12]. Researchers who study emerging adulthood have described samples of predominantly White college students in developed countries [56]. The unique aspects of emerging adulthood among marginalized individuals, such as former adolescent mothers, have not been well documented. We found that some mothers in our study described immense personal growth and maturation while they were parenting, while others were forced to give up attention to their own development to meet the demands of parenting. The personal growth of young mothers in this study was not directly reflected in the quantitative measures. Qualitative analyses revealed a more complex picture, with some mothers experiencing tremendous personal growth and maturation, and others describing experiences of growing alongside their child at a more measured pace. The reasons for these differences require further research.

Emerging adulthood also represents a period of social identity exploration, typically characterized by a shift from family members to friends and romantic partners for companionship and support [12,14]. Women who raise children through adolescence and emerging adulthood may have particularly complex support relationships with individuals in their social network, including their children’s fathers, their peers, and their own mothers or other family members [57]. Often, young mothers are not partnered with the father of their baby, and this relationship can be quite challenging [58,59], although more favorable maternal and child outcomes are generally seen when the mother and father are able to co-parent together [58,60].

Many young mothers live with their own family members, who often provide caregiving support as well as financial and emotional support [61], but given the multifactorial needs (e.g., caretaking, economic, housing) faced by a former adolescent mother, these relationships can become quite complicated. Researchers have reported the buffering effects of perceived social support from partners and the mother’s own family on parenting competency in samples of adolescent mothers [62,63]; this was also observed in the present study. In our study sample, mothers experienced varying sources and degrees of support–their own mothers, partners, fathers of the children, and MTB home visitors–and support was described as integral to their parenting confidence and their personal growth. Not all mothers in the sample described significant support structures, and even if they described positive parenting experiences, the lack of support highlighted the significant challenges and demands of parenting they confronted. This was also consistent with previous research that documented that lower levels of social support increased the risk for parenting stress and depression among adolescent mothers [64,65].

### Experiences of mothers from the MTB intervention and control groups

Our findings suggested several sustaining effects of the MTB intervention and highlight MTB mothers’ experiences of parenting through young adulthood. The quantitative findings demonstrated some of the enduring effects of MTB on parenting behaviors and child behaviors in the sample. In the qualitative interviews, participants who were enrolled in MTB shared vivid stories about the influences of MTB visitors on their ongoing parenting and their own growth from adolescence into emerging adulthood. In the data integration phase, we drew inferences about the sustaining effects of MTB by examining control and MTB group differences.

A central focus of the original MTB intervention was to help develop reflective capacities in young mothers to enhance their understanding of their infants and toddlers [11]. Reflective parenting relates to a parent or caregiver’s capacity to keep a child’s thoughts and feelings in mind and is a key factor in positive parenting [9,66]. In the ESA study, researchers found that women in the MTB group had lower levels of Prementalizing Modes (an inability to understand that a child’s behavior relates to their mental state or emotions) but no increase in Interest and Curiosity (interest and curiosity in the child’s thoughts and feelings; [9,16,67]. In this study, which included a younger cohort than the ESA study, there were no demonstrated MTB effects related to reflective parenting. However, our qualitative findings demonstrated that many MTB mothers were quite reflective about their children’s feelings, their parenting experiences, and their personal growth over time. Further research is needed with larger samples and perhaps more varied measures to gain further insight into reflective parenting among former adolescent mothers.

Researchers have demonstrated the positive effects of early home visiting in areas related to parent-child attachment, positive parenting, and developing a foundation for future parent and child health, particularly for parents who may be at risk for adverse child health and parenting outcomes [8,10,68,69]. Although our sample was small, our findings suggested that benefits of early home visiting and promotion of supportive parenting as a protective intervention may extend beyond the early childhood period. In the mixed methods analysis, we described key differences such that mothers in the control group had divergent responses to the challenges of parenting and child behavior problems, as some exhibited strength and others were overwhelmed. Mothers in the MTB group reported fewer problems yet still described immense challenges of parenting. However, MTB may have contributed to their increased confidence and patience in parenting, as many MTB mothers recalled what they had learned from their home visitors.

Metainferences highlighted lasting MTB effects on maternal Hostile/Coercive parenting and CBCL Total Problems, but also suggest questions for future research. Among adult samples, researchers have demonstrated that sensitive and positive parenting promotes more favorable outcomes for both the mother and child [70], highlighting the need for ongoing research to promote healthy parenting practices among former adolescent mothers. Additionally, maternal childhood adversity history is often associated with later negative parenting [71] and child behavior problems in samples of adult and adolescent mothers [49,72]. MTB mothers reported less hostile and coercive parenting and fewer child behavior problems; however, there were no differences in reported maternal early life adversity between the control and MTB groups. Despite the group differences, mothers in both groups described significant stress related to their children’s behavior problems, which supported the existing literature on parenting stress among adolescent mothers [46]. Future research is needed to further understand the complexities among early adversity history, protective factors, and positive parenting outcomes among former adolescent mothers.

There are limitations to this study. Although we detected statistically significant differences in the quantitative analysis, the sample size was small, and it is possible that clinically meaningful associations were missed. We were able to include equivalent numbers of control and MTB participants in the qualitative interviews and attempted to recruit a racially and ethnically diverse sample; nevertheless, it was ultimately likely that there was some selection bias in the participants who chose to respond (e.g., more enrolled participants reported being married or living with partner). It is possible that parenting experiences that were culturally related to race and ethnicity were not fully explored in the qualitative phase as we were unable to differentially enroll MTB and control participants based on these social characteristics. The PI was not blinded to the group status of participants throughout the qualitative data collection and analysis phase. To bracket during data collection and analysis, the PI used memos to reflect on potential biased differential outcomes between the two groups. Considerations of racial discordance in research are important [73]; in this study the PI is White, while most of the qualitative sample self-identified as Black and Hispanic. The qualitative interview included questions about sensitive topics, and it is possible that racial discordance may have affected responses. Lastly, although the use of Zoom interviews may have enhanced convenience and participation rates, the sample may also have been biased towards those who had ready access to technology and internet access.

## Conclusion

This mixed methods study offers insights into the complex parenting and intersectional developmental experiences among a cohort of women who had a first birth in adolescence. Since this study sample included women enrolled in a clinical trial testing a reflective parenting EHV program, we were able to describe some of the longer-term program effects that persisted past the end of the intervention and into the demanding developmental stage of emerging adulthood. Despite the many stressors of adolescent parenthood, mothers in our study revealed steadfast dedication to love, nurture, protect, and care for their children. The qualitative data included powerful stories illuminating the challenges faced and met by the participants. Integrated findings inform clinical and research approaches to promote health equity, personal growth and development, and positive parenting outcomes, during the phases of emerging and early adulthood, among women who began childbearing in adolescence.

## Supporting information

S1 Table ESA study variables and measures for use in secondary analysis. (PDF)

S2 Table Semi-structured interview guide. (PDF)

S3 Table COREQ (Consolidated criteria for Reporting Qualitative research) checklist. (PDF)

S4 Table Study variables. (PDF)

S5 Table Spearman correlations between maternal experiences and parenting outcomes. (PDF)
