# Supplementary material for: Parenting experiences and outcomes among former adolescent mothers: A mixed methods study
Source: PLoS One. 2024 May 15;19(5):e0303119. doi: 10.1371/journal.pone.0303119 (PMC11095697; doi:10.1371/journal.pone.0303119)
Supplement: S1 Table — (PDF) [file pone.0303119.s001.pdf]

S1 Table. ESA Study Variables and Measures for Use in Secondary Analysis

Theory	Concept	Variable	ESA Follow-up Cohort Study Measure	Cronbach's alpha for ESA Measures
Belsky's Determinants of Parenting (Maternal Experiences)	Maternal early life experiences	Maternal early life adversity and family strengths	Childhood Trauma Questionnaire (CTQ)	α =0.95
	Maternal trauma history	Post-Traumatic Stress Disorder (PTSD) symptoms	PTSD Checklist – Civilian Version (PCL-C)	α =0.97
	Developmental and life course indicators	Educational status<br>Employment<br>Marital status<br>Maternal age<br>Pregnancy spacing	Demographic survey	
Belsky's Determinants of Parenting (Parenting Outcomes)	Parental reflective capacity	Reflective functioning	Parental Reflective Functioning Questionnaire (PRFQ)	α =0.65 (PRFQ-IC)<br>α =0.70 (PRFQ-PM)
	Parenting behaviors	Parenting behaviors (Supportive/ Engaged, Hostile/Coercive)	Parenting Behavior Inventory	α =0.69 (Supportive/Engaged)<br>α =0.69 (Hostile/Coercive)
	Maternal report of child behavior	Child behavior and emotional problems	Child Behavior Checklist (CBCL)	Children ≤ 5 years:<br>α =0.91 (Total)<br>α =0.84 (Externalizing)<br>α =0.76 (Internalizing)<br><br>Children ≥6 years:<br>α =0.97 (Total)<br>α =0.97 (Externalizing)<br>α =0.84 (Internalizing)

Note. PRFQ-IC is Interest and Curiosity subscale, and PRFQ-PM is Prementalizing Modes subscale.
