# Supplementary material for: Parenting experiences and outcomes among former adolescent mothers: A mixed methods study
Source: PLoS One. 2024 May 15;19(5):e0303119. doi: 10.1371/journal.pone.0303119 (PMC11095697; doi:10.1371/journal.pone.0303119)
Supplement: S2 Table — (PDF) [file pone.0303119.s002.pdf]

S2 Table. Semi-Structured Interview Guide.

Topic or Associated Quantitative Variable	Question and Prompts
Warm-up	1. Could you tell me about your family? I'd love to hear about the family and the people you're living with now.<br>How many children do you have? What are their ages?
Experiences of parenting	2. Can you tell me about your story of becoming a mother, starting at the beginning when you first found out you were pregnant through to today?<br>*Prompt:*<br>a. When you were a part of Minding the Baby®, you were a teenager. Can you describe yourself as a parent?<br>b. How has your parenting changed over time from when you first became a mother to now?
Intersectionality<br>Experiences of parenting	3. Tell me about other people (father of the baby, other family members, other friends or community members) who been involved in your parenting.<br>*Prompt:*<br>a. Who has been most helpful to you and how? Can you describe how this person supported your parenting?<br>b. Who has been least helpful to you and why? Can you describe how this person may have affected your parenting?<br>c. Do you have any cultural traditions or family traditions for childrearing or parenting that have helped you?
Parenting in early adulthood<br>PTSD (4c)	4. How has being a parent influenced your life, particularly as you have parented through your early adulthood?<br>*Prompt:*<br>a. Can you tell me a bit about how you think parenting has affected some of the choices and decisions you have made for yourself as you've moved into adulthood?<br>b. If you could do it all over again, is there anything you would change about how you have parented your child(ren) or would do differently?<br>c. Are there any areas of stress that you're experiencing now that are affecting your life or your parenting?

PRFQ	5.	You may need to think about this question. Please take your time. Parenting can be very rewarding. When you think about your role as a parent, can you tell me about a time when you felt proud or happy as a mother?<br>*Prompt:*<br>a. Can you tell me a story that feels memorable to you?<br>b. Can you think of a time when you were curious about how your child was feeling (or a time when you found yourself thinking about how your child might be feeling)?
PBI	6.	Again, please take your time answering this question. Parenting can also sometimes be very challenging. What have been some of the most difficult parts of parenting for you?<br>*Prompt:*<br>a. Can you describe a particular time when you found your experience as a parent to be really hard? How did you respond to your child during that time?<br>b. How do you deal as a parent with the moments in parenting that are hard?
CBCL	7.	Can you describe your child now?<br>*Prompt:*<br>a. What are his/her moods like? Do you have any concerns?<br>b. Does your child experience any acting out? Do you have any concerns about his/her behavior?<br>c. What is your relationship like with your child now?
Group status/intervention	8.	<u>For MTB early home visiting intervention participants only:</u><br>You were a part of Minding the Baby® when you were a new mother. Can you tell me why you wanted to be a part of the program?<br>*Prompts:*<br>a. Can you tell me about what you remember from when you first started Minding the Baby®?<br>b. Are there any visits or things that you learned about parenting that you remember specifically?<br>c. What would you say was most helpful about Minding the Baby® for you? What (if anything) have you been able to use from what you learned in Minding the Baby® to help you parent as your child grew and developed….as you learned to be a parent of an older child?<br><br><u>For control participants only:</u>

		You were a part of the Minding the Baby® study when you were a new mother. Can you tell me why you wanted to be a part of the research study?<br>*Prompts:*<br>d. Can you tell me about what you remember from the research study?<br>e. Are there any particular research visits that you remember?
Intersectionality, COVID, racism	9.	This past year and a half has been particularly stressful for a lot of parents, for many different reasons. One ongoing stressor has been the COVID-19 pandemic. Can you describe what parenting has been like for you during COVID?<br>*Prompt:*<br>a. Can you describe your experience of the pandemic and how it has affected you and your family, and specifically, how it has affected your parenting?<br><br>Our country has also faced a reckoning with systemic racism and experiences of racism and injustices towards people of color. As I mentioned, I am a pediatric nurse, and I'm interested in learning about experiences of individuals and families living in different communities in New Haven. You said that you identify as [race/ethnicity identification in demographic survey], so as a member of that community, can you tell me about your experiences (as a [how participant identifies]) living in your community?<br>*Prompt:*<br>a. Can you tell me how the racial and social justice movements have, if at all, affected your experiences as a parent? Or as a young adult living in New Haven?
PRFQ	10.	When you think about the next 5 years, what are three wishes or goals you have for yourself as a parent?<br><br>When you think about the next 5 years, what goals do you have for yourself as a person?<br><br>And for your child(ren)?
Qualitative methods (Zoom interviewing among young adults)	11.	Having interviews over Zoom is new for all of us. How was this experience for you? Can you tell me a little about it?

Closing	12.	Is there anything I have missed or anything else you would like to tell me?<br>Thank you very much!
