# Supplementary material for: Parenting experiences and outcomes among former adolescent mothers: A mixed methods study
Source: PLoS One. 2024 May 15;19(5):e0303119. doi: 10.1371/journal.pone.0303119 (PMC11095697; doi:10.1371/journal.pone.0303119)
Supplement: S4 Table — (PDF) [file pone.0303119.s004.pdf]

S4 Table. Study Variables.

Collected During ESA Follow-up Study October 2016 to March 2018.

	Total (*N*=71)	Control Group (*n*=39)	Intervention Group (*n*=32)	
Variable	Mean (*SD*)	Mean (*SD*)	Mean (*SD*)	*p* value
Childhood Trauma Questionnaire (CTQ) Total Score	40 (18.1)	43.3 (18.4)	37.3 (17.4)	.09
The PTSD Checklist-Civilian Version (PCL-C) Total Score	32.6 (15.3)	33.9 (15.8)	31.1 (14.7)	.35
Parental Reflective Functioning Questionnaire (PRFQ)				
Interest/Curiosity in Mental States (PRFQ-IC)[a]	34.1 (6.3)	34.3 (6.3)	34.0 (6.4)	.89
Parenting Behavior Inventory (PBI)				
Hostile/Coercive Parenting[b]	12.4 (5.5)	13.9 (5.9)	10.6 (4.4)	.009**
Child Behavior Checklist (CBCL)				
Total Problems[b]	49.9 (11.6)	52.2 (12.4)	47.1 (10.1)	.06
Externalizing Behaviors[c]	48.4 (11.5)	51.0 (12.4)	45.3 (9.6)	.04*
Internalizing Behaviors[b]	49.9 (10.8)	51.6 (10.7)	47.9 (10.8)	.03*

Variable Dichotomized at Median	n (%)	n (%)	n (%)	*p* value
Prementalizing Modes (PRFQ-PM)[d]				
Higher (more impaired mentalizing)	31 (44)	26 (67)	16 (52)	.20
Lower (less impaired mentalizing)	39 (56)	13 (33)	15 (48)	
Supportive Parenting (PBI-supportive)[d]				
Higher (more supportive parenting)	32 (45)	21 (54)	19 (60)	.64
Lower (less supportive parenting)	39 (55)	18 (46)	13 (41)	

Note.

[a] Variable was cube transformed for t-test, but reported means and SD are raw numbers.

[b] Independent samples t-test; Mann-Whitney U for all other continuous variables.

[c] Variable was log transformed for t-test, but reported means and SD are raw numbers.

[d] Pearson's chi-square test use for this variable.

*p < .05, **p < .01, ***p < .001.
