# Supplementary material for: Parenting experiences and outcomes among former adolescent mothers: A mixed methods study
Source: PLoS One. 2024 May 15;19(5):e0303119. doi: 10.1371/journal.pone.0303119 (PMC11095697; doi:10.1371/journal.pone.0303119)
Supplement: S5 Table — (PDF) [file pone.0303119.s005.pdf]

S5 Table. Spearman Correlations Between Maternal Experiences and Parenting Outcomes.

Sample	Measures	PRFQ-IC	PRFQ-PM	PBI Hostile/Coercive	PBI Supportive/Engaged	CBCL Total Problems	CBCL Externalizing Behaviors	CBCL Internalizing Behaviors
Total (N=71)	CTQ Total Score	-.05	.46***	.02	-.35**	.53***	.46***	.44***
	PCL-C	.19	.41***	.19	-.21	.61***	.46***	.59***
Control (n=39)	CTQ Total Score	-.18	.66***	.10	-.43**	.53***	.49**	.45**
	PCL-C	.20	.66***	.21	-.37*	.68***	.53***	.63***
Intervention (n=32)	CTQ Total Score	.08	.17	-.23	-.22	.53**	.43*	.40*
	PCL-C	.18	.06	.16	-.04	.59***	.39*	.53**

Note. CTQ Total Score and PCL-C are maternal experiences, and PRFQ, PBI, and CBCL are parenting outcomes.
*$p < .05$, **$p < .01$, ***$p < .001$.
